# Characteristics of intramedullary nail breakage in pertrochanteric femur fractures: a summary of 70 cases

**DOI:** 10.1186/s13018-021-02826-3

**Published:** 2021-11-17

**Authors:** Pengfei Li, Zhishan Zhang, Fang Zhou, Yang Lv, Yan Guo, Yun Tian

**Affiliations:** 1 Department of Orthopaedics, Peking University Third Hospital, No. 49 North Huayuan Road, Haidian District, Beijing, 100191 People’s Republic of China; 2 Center of Foot and Ankle Surgery, Beijing Tongren Hospital, Capital Medical University, No. 1 Dongjiaomin Lane, Dongcheng District, Beijing, 100730 People’s Republic of China

**Keywords:** Pertrochanteric fractures, Unstable fractures, Intramedullary nail breakage, Revision surgery, Self-dynamisation

## Abstract

**Objective:**

To reveal noteworthy characteristics of intramedullary (IM) nail breakage in pertrochanteric femur fractures.

**Materials and methods:**

The data from 6 patients with IM nail breakage in our hospital between August 2008 and May 2018 were reviewed retrospectively. With an additional 64 cases reported in articles in the PubMed database prior to October 2019, a total of 70 cases were reviewed and analysed; epidemiological patient data and data on the initial trauma, fracture type, nail selected for the original surgery, time from surgery to breakage, mechanism and location of breakage, status of fracture healing, salvage treatment and prognosis were assessed.

**Results:**

Seventy patients with pertrochanteric fractures, including 2 stable fractures and 68 unstable fractures, experienced IM nail breakage at a mean of 9.4 months after the initial surgery. Among them, 9 (12.9%) occurred within 3 months, 23 (32.9%) between 3 and 6 months and 38 (54.3%) after 6 months. The mean age was 72.3 years, and 16 (22.9%) patients were younger than 65 years old. When nail breakage occurred, 66 fractures (94.3%) exhibited delayed union/non-union. Self-dynamisation was found in 12 cases (17.1%). The salvage procedures included 4 partial/total implant removal procedures, 17 hemi/total hip arthroplasty procedures, 3 conservative treatment procedures, and 46 revised osteosyntheses, of which 7 cases (15.2%) sustained secondary implant failure. No significant differences were found between the failure rates of IM nails and extramedullary(EM) devices (odds ratio [OR], 3.429; 95% confidence interval [CI], 0.632–18.877; *p* = 0.330).

**Conclusion:**

IM nail breakage is a rare complication lack of time regularity and mostly occurs in unstable pertrochanteric fractures in the presence of delayed union/non-union. Osteosynthesis revision can be conduct by a new IM nail or EM device but considerable secondary failure rate is noteworthy. Self-dynamisation may be a warning sign of nail breakage.

## Introduction

Pertrochanteric femoral fractures are becoming increasingly common with population ageing and are usually treated surgically by internal fixation. Intramedullary (IM) nails are now the most widely used and recommended devices for pertrochanteric fractures, especially the unstable ones [1, 2]. However, implant failure for IM nails remains a problem for pertrochanteric femoral fractures, despite the existence of improved techniques and various implant modifications [3].

Nail breakage is a rare form of mechanical failure of the implant but may result in substantial disability and extra medical costs for the patient; this complication is usually reported as a single case or within a small cohort due to the low incidence [4, 5]. Thus, a series of questions still remain, including when, where, and in which type of fractures and which devices breakage tends to occur, as well as which management and prevention strategies are optimal for this form of mechanical failure.

Therefore, the purposes of this retrospective study were to report a case series of nail breakage in pertrochanteric femur fractures treated at our institution and to reveal the characteristics of this kind of implant failure by reviewing both these cases and previous cases in the literature.

## Materials and methods

### Patients treated at our centre

The medical records of 785 consecutive adult patients who underwent surgery with intramedullary nails for trochanteric femur fractures at our hospital between August 2008 and May 2018 were retrospectively reviewed. We also reviewed the medical records and radiologic data of patients referred to our hospital for salvage surgery due to implant failure. The inclusion criterion was the presence of intramedullary nail breakage. The exclusion criteria were as follows: (1) a pathological fracture, delayed fracture (> 2 weeks), atypical fracture, sub-trochanteric fractures or periprosthetic fracture at the initial operation, (2) initial treatment with extramedullary (EM) devices, or (3) < 12 months of follow-up data.

### Literature review and cases screening

A literature search of the PubMed database for articles published prior to October 31, 2019 was performed using the following strategy: (“nail breakage” OR “nail fracture” OR “nail rupture” OR “broken nails”) AND ((hip fractures [mesh] NOT femoral neck fractures [mesh]) AND intramedullary nails). Potentially eligible studies were required to meet the following inclusion criteria: the patients had trochanteric femur fractures, IM nails were used to treat the fractures, nail breakage was subsequently confirmed, and detailed information for at least one case was reported. Then, the reference lists of the articles included were manually screened to identify additional relevant studies meeting the criteria above.

All cases were screened, and the exclusion criteria were as follows: (1) a pathological fracture, delayed fracture (> 2 weeks), atypical fracture, sub-trochanteric fractures or periprosthetic fracture at the initial operation; and (2) missing data on the broken nail, implant in the salvage procedure or prognosis.

### Patient data and statistical analysis

The epidemiological patient data and data on the initial trauma, fracture type, nail selected for the original surgery, time to implant rupture, mechanism of nail breakage, location where the nail failed, status of fracture healing, salvage treatment and prognosis were recorded.

The fracture types in the patients at our institution were classified following the AO/OTA classification system [6] by two orthopaedists collaboratively on the basis of the preoperative anteroposterior X-rays of the hip. The fracture types of the patients reported in previous studies were determined on the basis of the information provided by the authors or by two orthopaedists collaboratively on the basis of the radiographs provided in the original paper if the AO/OTA classification system was not used.

SPSS version 18.0 (Chicago, IL, USA) was used to perform the analysis. Continuous and categorical parameters were analyzed by Mann–Whitney *U* test and the chi-squared test respectively. Fisher’s exact test was used for small data subsets (*n* < 5). *P* < 0.05 was considered statistically significant.

## Results

### Report of 6 cases

Six cases (Table 1) were identified; 3 cases underwent initial surgery in our hospital, and 3 underwent surgery in other hospitals. The rate of intramedullary nail breakage in our centre was 0.38% (3 in 785).

**Table.1:** Broken nails in our hospital (*n* = 6)

Nos.	Age/sex	Mechanism of initial trauma	AO/OTA classification	Initial implants	Months until breakage	Site	Mechanism of nail breakage	Fracture healing	Revision implants
1	69/M	Traffic accident	31 A3	Long InterTan	10	Proximal aperture	Atraumatic	Non-union	PFP
2	79/M	Simple fall	31 A3	Short PFN	84	Proximal aperture	During implant removal	Union	Partial implant removal
3	80/M	Simple fall	31 A3	Short PFNA2	10	Proximal aperture	Atraumatic	Non-union	PFP
4	49/M	Fall > 2 m	31 A3	Short PFNA	21	Proximal aperture	Fall > 2 m	Union	Implant removal
5	66/F	Simple fall	31 A3	Long InterTan	10	Proximal aperture	Atraumatic	Non-union	THA
6	41/M	Traffic accident	31 A2	Short PFNA	9	Proximal aperture	Atraumatic	Non-union	Long InterTan

PFN, proximal femoral nail; PFNA, proximal femoral nail antirotation; PFNA2, proximal femoral nail antirotation for Asia; PFP, proximal femur locking plate; THA, total hip arthroplasty

One of these patients was a woman, and the remaining 5 were men, with a mean age of 64.0 years (range 41–80 years) at the time of initial surgery. All fractures were unstable, including 1 31 A2 fracture and 5 31 A3 fractures; 3 of them were caused by high-energy injuries. The broken nails included 1 short PFN, 1 short PFNA2, 2 short PFNAs and 2 long InterTan nails. All nails broke at the proximal aperture.

Two devices broke in pertrochanteric fracture cases initially exhibiting union: a short PFNA nail broke at 21 months due to a horse rider’s second fall, and a short PFN broke at 84 months during implant removal, which was performed based on the patient’s needs. The short PFNA was completely removed, while only the proximal part of the PFN was removed due to surgical difficulty and with the consent of the patient’s family.

The other 4 nails broke when the patients suffered sudden, atraumatic hip pain at 9 or 10 months after the first surgery. All fractures exhibited non-union; 2 of them were revised with a proximal femur locking plate (PFP), one was revised with a long InterTan nail, and one was revised with total hip arthroplasty. The 3 fractures that underwent revision osteosynthesis finally exhibited union, and all 4 patients could walk normally without assistance at the 12-month follow-up.

### Analysis of 70 cases

Senventy cases were analysed, including the 6 cases above and 64 cases reported in 19 previous papers [4, 5, 7–23] (Table 2). The mean age of the included patients was 72.3 years old (range 35–94 years), and 16 (22.9%) patients were younger than 65 years old. There were 36 males, 25 females and 9 patients of an unknown sex. A total of 18.6% of the patients (13 in 70) suffered from high-energy injuries, and 97.1% of them (68 in 70) had unstable fractures, including 24 31 A2 fractures and 44 31 A3 fractures.

**Table.2:** Previous studies and matched cases

Broken nails (*n* = 64)	Papers (*n* = 19)
13	Tomás-Hernández (2018)
11	Lambers (2019) and von Rüden (2015)
8	Cruz-Sánchez (2015)
4	Álvarez (2004)
2	Kasimatis (2007), Rappold (2001) and Gaebler (1999)
1	Rollo (2018), Zheng (2017), Sbiyaa (2016), Giannoudis (2013), Iwakura (2013), Maniscalco (2013), Wee (2009), Karladani (2006), Yoshino (2006), Wozasek (2002) and Van de Brink (1995)

**Fig. 1:**
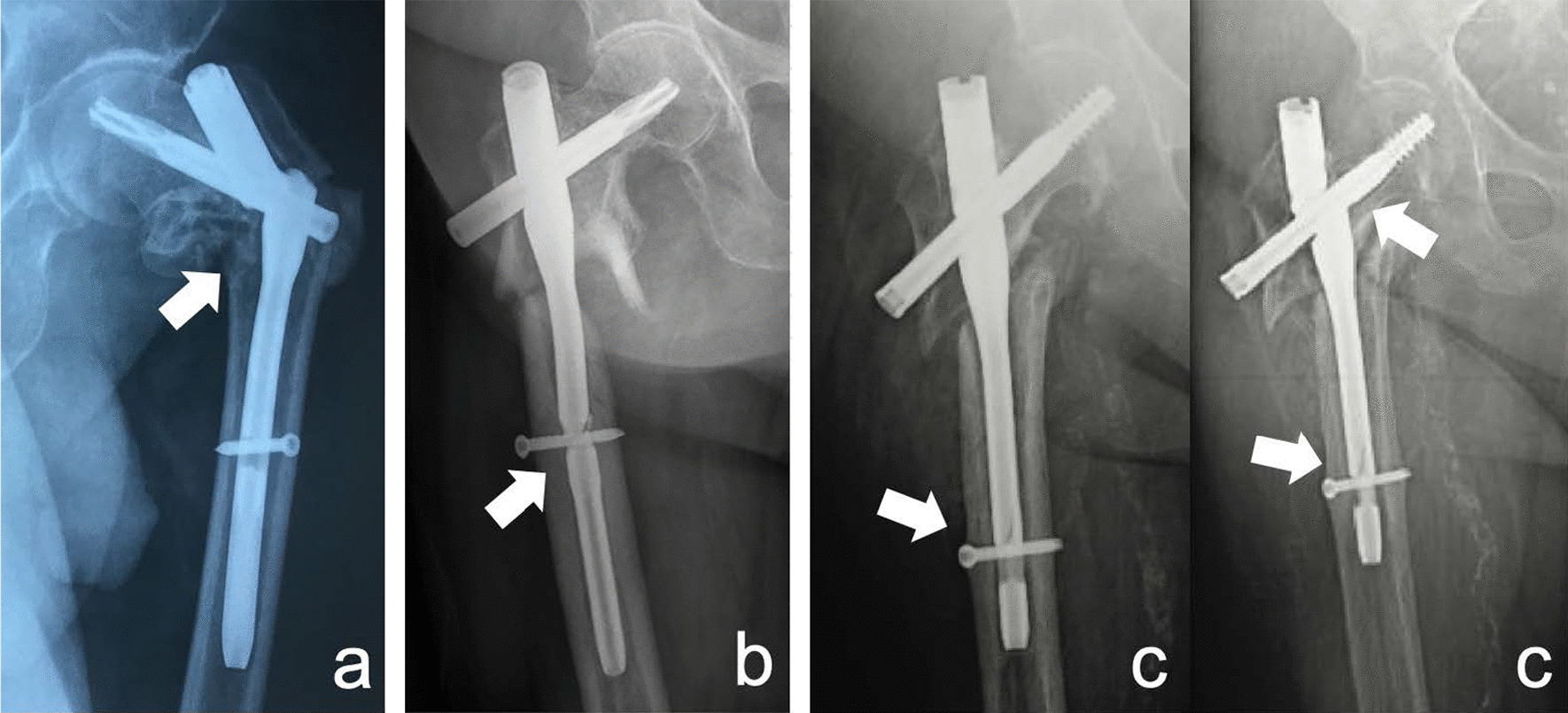
Location at which nail breakage occurs. **a** An 80-year-old male with a 31 A3 fracture was treated with a short PFNA2, and the nail broke at the proximal aperture 10 months later. **b** A 77-year-old female with a 31 A3 fracture was treated with a short TFN, and the nail broke at the distal aperture 2 months later. **c** An 83-year-old female with a 31 A3 fracture was treated with a short Affixus nail, and nail breakage occurred at the distal aperture and proximal aperture sequentially after 1 and 2 years, respectively

Forty-seven short nails and 23 long nails broke at a mean of 9.4 months (range 1–84 months) after osteosyntheses (Tables 3, 4). A total of 87.1% of the breakage cases (61 in 70) were atraumatic, 5 of them were due to re-trauma, and 4 of them were found at implant removal. Sixty-one nails broke at the proximal aperture, 7 nails broke at the distal aperture, 1 nail broke at the proximal and distal apertures simultaneously, and 1 nail broke at the proximal aperture 1 year after an unprocessed breakage at the distal aperture (Fig. 1). When nail breakage occurred, 65 fractures (92.9%) exhibited delayed union/non-union. Self-dynamisation was found in 12 cases (17.1%).

**Table.3:** Clinical characteristics of the patients involved in the study (*n* = 70)

Parameter	Value
Age (years) (mean, min–max)	72.3 (35–94)
< 65 (*n*, %)	16 (22.9%)
≥ 65 (*n*, %)	54 (77.1%)
Sex (*n*, %)	
Female	36 (51.4%)
Male	25 (35.7%)
Unknown	9 (12.9%)
Initial trauma (*n*, %)	
Low energy (simple fall)	57 (81.4%)
High energy	13 (18.6%)
AO/OTA classification (*n*, %)	
31 A1	2 (2.9%)
31 A2	24 (34.3%)
31 A3	44 (62.9%)
Broken nails (*n*, %)	
Short	47 (67.1%)
Long	23 (32.9%)
Time from surgery to breakage (months) (mean, min–max)	9.4 (1–84)
≤ 3 (*n*, %)	9 (12.9%)
3–6 (*n*, %)	23 (32.9%)
> 6 (*n*, %)	38 (54.3%)
Mechanism of nail breakage (*n*, %)	
Atraumatic	61 (87.1%)
Traumatic	5 (7.1%)
Iatrogenic	4 (5.7%)
Site (*n*, %)	
Proximal aperture	61 (87.1%)
Distal aperture	7 (10.0%)
Proximal and distal apertures	2 (2.9%)
Fracture healing when breakage (*n*, %)	
Non-union/delayed union	65 (92.9%)
Infected non-union	1 (1.4%)
Union	4 (5.7%)
Self-dynamism before breakage (*n*, %)	
Yes	12 (17.1%)
No	58 (82.9%)
Management (*n*, %)	
Osteosynthesis revision	46 (65.7%)
Arthroplasty	17 (24.3%)
Implant removal	4 (5.7%)
Conservation	3 (4.3%)

**Table.4:** Type of broken nails (*n* = 70)

Implants			Total (%)
Type	Short (*n*)	Long (*n*)	
Gamma	24	12	36 (51.4%)
InterTan	5	6	11 (15.7%)
TFNA	6	4	10 (14.3%)
PFN	4	0	4 (5.7%)
PFNA	3	0	3 (4.3%)
TFN	1	1	2 (2.9%)
PFNA2	1	0	1 (1.4%)
AFFIXUS	1	0	1 (1.4%)
Endovis	1	0	1 (1.4%)
IMHS	1	0	1 (1.4%)
Total	47	23	70 (100%)

Among the 70 patients, 4 underwent partial/total implant removal, 17 underwent hemi/total hip arthroplasty, and 3 chose conservative treatment. Forty-six patients underwent revised osteosyntheses, and 7 of them (15.2%) sustained secondary implant failure, including 4 of the 36 IM nail cases (11%) and 3 of the 10 EM device cases (30%). No significant differences were found between the re-failure rates of IM nails and extramedullary devices (odds ratio [OR], 3.429; 95% confidence interval [CI], 0.632–18.877; *p* = 0.330) (Table 5).

**Table.5:** Results of the implants used in osteosynthesis revision (*n* = 46)

Revised implants	Implant failure		Failure rate (%)	Odds ratio (95%CI)	*p* value
	No	Yes			
	*n* (%)	*n* (%)			
EM device	7 (18)	3 (43)	30	3.429 (0.623–18.877)	0.330
IM nail	32 (82)	4 (57)	11		
Total	39 (100)	7 (100)	15		

## Discussion

IM nail breakage in pertrochanteric fractures is a rare complication that usually occurs in unstable cases [3], the prevalence of this complication has been reported to be 0.87–0.88% in previous studies [7, 11]. Some authors found the incidence to be high, ranging from 2.9 to 5.7%, while they included both pertrochanteric fractures and subtrochanteric fractures [10, 13, 21]. Here, we report a case series of 6 pertrochanteric fractures with nail breakage. The rate of IM nail breakage in our centre was 0.38% (3 in 785), which is in accordance with the results in previous studies and confirms the frequency of this complication to be low in pertrochanteric fractures [7, 11].

**Fig. 2:**
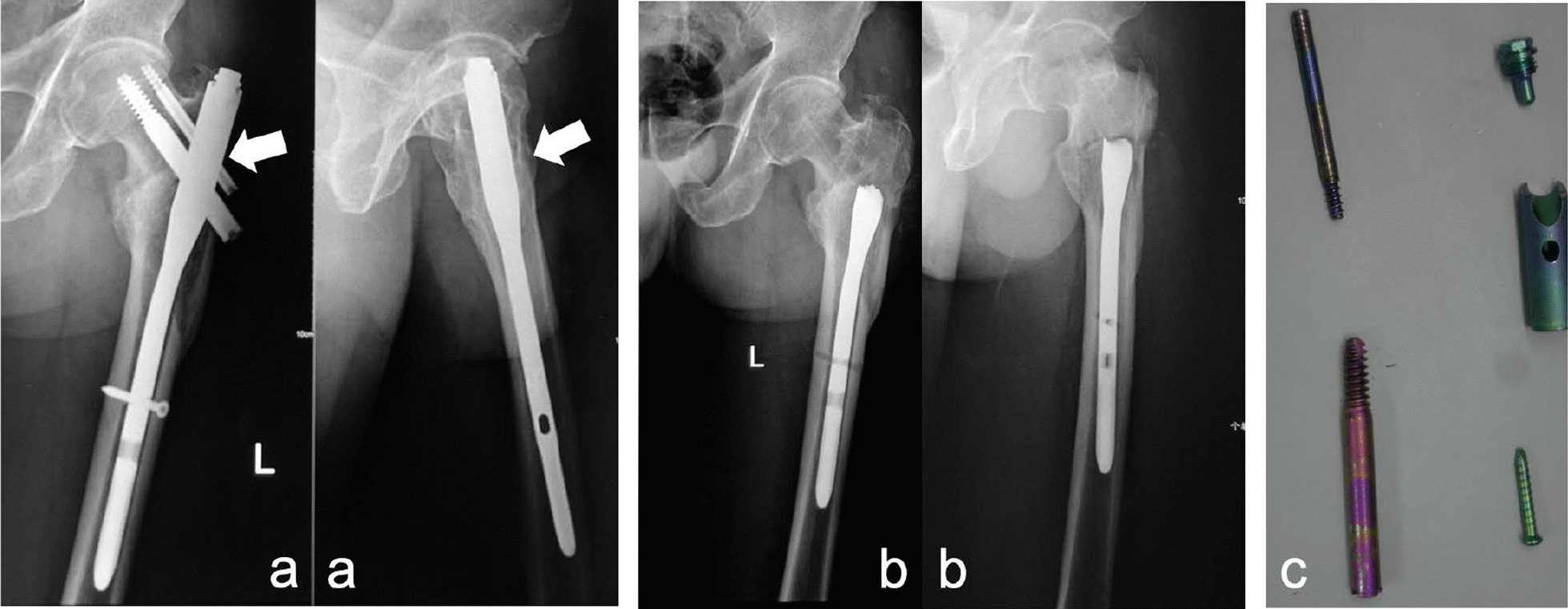
A 79-year-old male with a 31 A3 fracture was treated with a short PFN, and the fracture was healed. The patient complained of progressive discomfort in the lateral hip 4 years after the initial operation and decided to undergo removal of the PFN 3 years later. **a** No signs of nail breakage were observed on the X-rays before implant removal. **b**, **c** Nail breakage was found during the operation, and only part of it was removed to avoid iatrogenic injury

Among the 70 cases included, nail breakage occurred in most (92.9%) cases in pertrochanteric fractures that did not exhibit union because “fracture healing is a race between bony union and implant failure”. However, there were still 4 cases in which nail breakage and bony union were both observed, 2 in our study and 2 in previous studies [10, 17]. We hypothesize that the IM nails were broken incompletely or completely but without displacement when the fractures achieved bony union and were detected by X-ray when displacement appeared due to trauma on the ipsilateral limb or found by orthopaedists unexpectedly during elective implant removal [10, 17] (Fig. 2).

Though the presence of complex unstable fractures, suboptimal reduction, the unsatisfactory placement of IM nails and the occurrence of delayed union/non-union are the recognized reasons for nail breakage [7, 10, 11, 14], these factors cannot explain why IM nails break rather than exhibit cut-out, a more common form of implant failure [3]. Johnson et al. [24] reported that a low American Society of Anaesthesiologists (ASA) score, subtrochanteric fracture and pathological fracture were independent risk factors for nail fractures, and the authors further proposed that young patients with a low ASA score are at the highest risk of nail breakage. Similarly, among the 70 patients included in this study, 16 (22.9%) patients were younger than 65 years old. We hypothesize that a younger age may be associated with not only more loading cycles postoperatively but also enough bone stock in the proximal femur to prevent cut-out; thus, when delayed union/non-union occurs, IM nails break. The design of different kinds of IM nails may also be a factor for nail breakage. A biomechanical experiment showed that the structure around the proximal aperture is a weak point of the gamma nail and can be damaged by improper drilling [10]. This finding may explain why breakage usually occurs at the proximal aperture level. Lambers et al. [4] reported a cohort of 13 patients and 16 broken TFNAs with unique stepped propagation of the implant fracture pathway in the early stage. The authors attributed this finding to changes in the prosthetic design of the TFNA and suggested that close clinical and radiographic surveillance is performed for patients with unstable hip fracture patterns undergoing osteosynthesis with a TFNA implant.

**Fig. 3:**
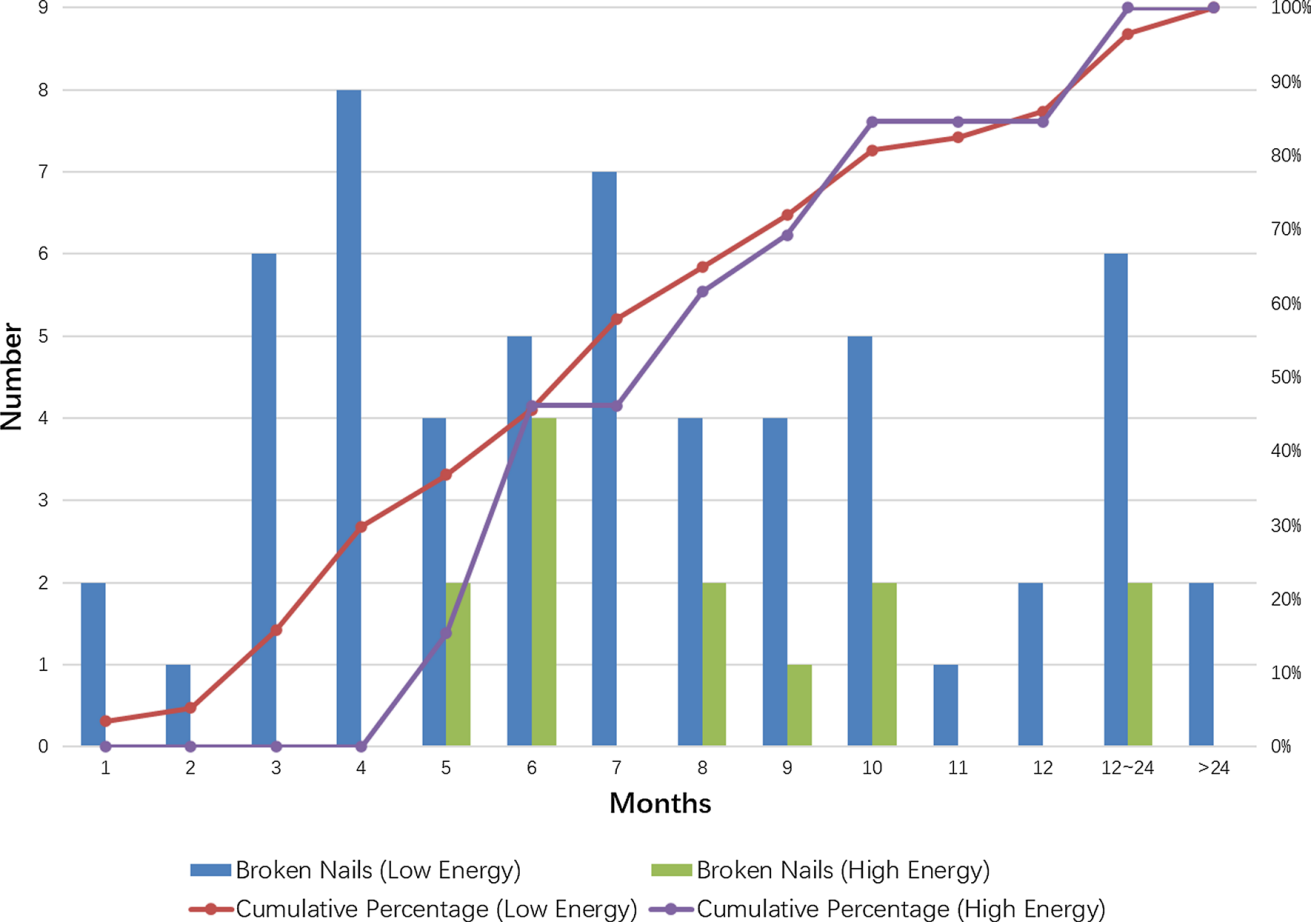
Number of cases according to the interval from the initial operation to nail breakage

Compared with cut-out, a common form of implant failure mostly occurs within 3 months postoperatively [25], IM nail breakage has a relatively late average occurrence time, but lacks of time regularity (Table 3; Fig. 3). Among the 70 patients included in this study, 9 (12.9%) nail breakage occurred within 3 months, 23 (32.9%) between 3 and 6 months and 38 (54.3%) after 6 months (Table 3). Thus, we recommend a routine follow-up for all patients for 6 months and an additional enhanced follow-up for patients with clinical signs of delayed union.

Pertrochanteric fractures caused by high energy trauma are more common in non-elder population, difficult to reduce, and more prone to complications [26, 27]. In 70 fractures included, 13 were caused by high energy trauma. Compared with low-energy group, these patients had more men (OR 0.126; 95% CI 0.038–0.680; *p* = 0.019) and a younger average age (54.8 ± 13.3 vs 76.4 ± 11.2; *p* = 0.0001) (Table 6), but there was no difference in fracture type, short/long nail selection and the occurrence of self-dynamisation. The average time from initial surgery to nail breakage of low-energy group and high-energy group were 9.4 ± 12.4 and 9.3 ± 5.7 months respectively but no nail breakage occurred less than 4 months in high-energy group (Fig. 3).

**Table.6:** Differences between patients with initial trauma of LOW OR HIGH energy (*n* = 70)

Parameters	Initial trauma		Odds ratio (95%CI)	*p* value
	Low energy (*n* = 57)	High energy (*n* = 13)		
Age(years) (mean, min–max)	76.4 ± 11.2	54.8 ± 13.3		0.0001
Gender (*n*, %)				
Female	33 (57.9%)	3 (23.1%)		
Male	16 (28.1%)	9 (69.2%)	0.162 (0.038–0.680)	0.019
No data	8 (14.0%)	1 (7.7%)		
AO/OTA classification (*n*, %)			1.414 (0.388–5.153)	0.834
A1&A2	22 (38.6%)	4 (30.8%)		
A3	35 (61.4%)	9 (69.2%)		
Broken nails (*n*, %)			0.889 (0.242–3.262)	1.000
Short	38 (66.7%)	9 (69.2%)		
Long	19 (33.3%)	4 (30.8%)		
Time from surgery to breakage (months) (mean, min–max)	9.4 ± 12.4	9.3 ± 5.7		0.318
Self-dynamism before breakage (*n*, %)			1.600 (0.367–6.984)	0.825
Yes	9 (15.8%)	3 (23.1%)		
No	48 (84.2%)	10 (76.9%)

**Fig. 4:**
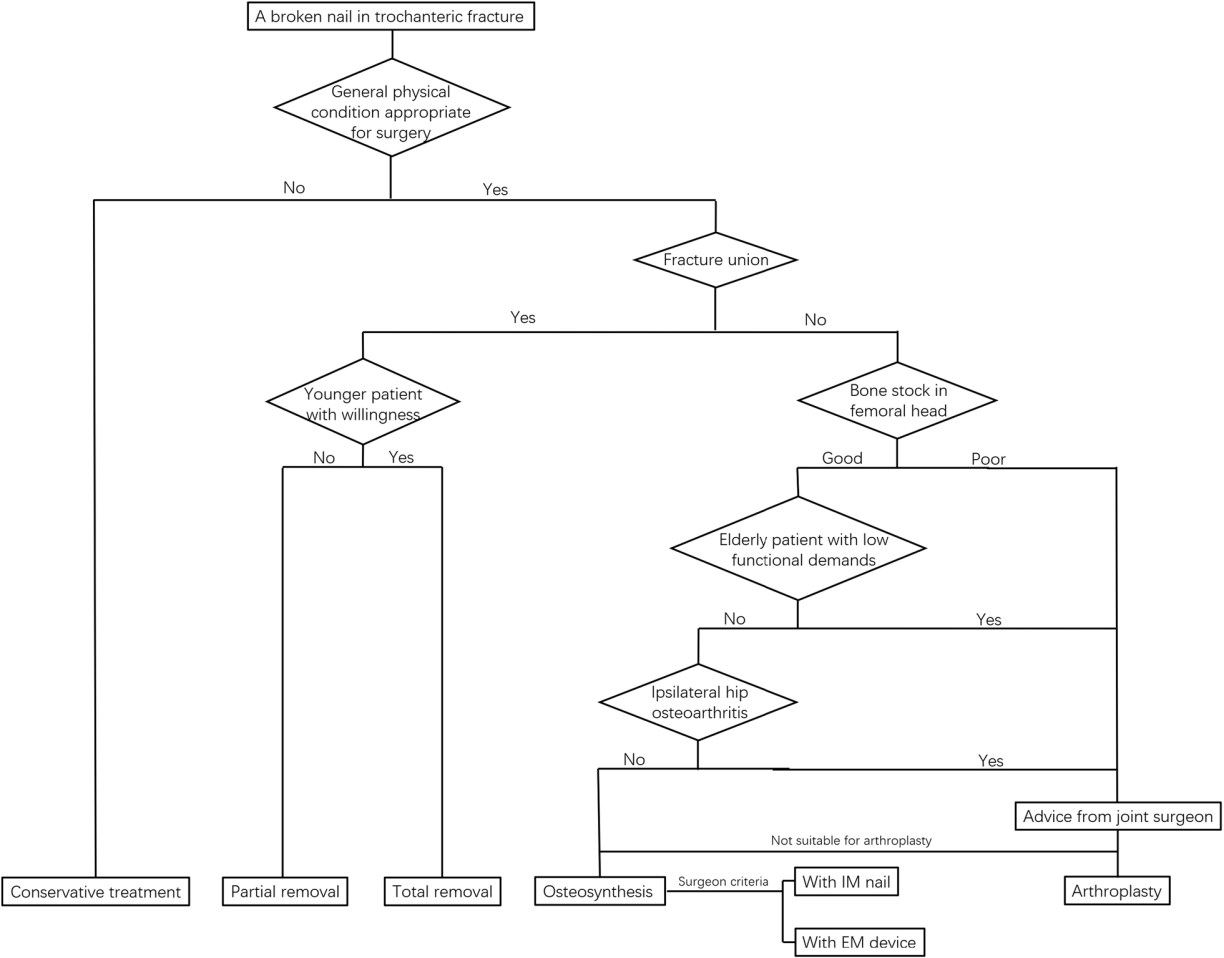
A recommended strategy to address nail breakage

The decision of whether to perform revision surgery to replace a broken nail should be made considering the patient’s individual characteristics. The following factors need to be considered by orthopaedic surgeons, as Tomas-Hernandez et al. [7] recommend in their article: the type of previous fracture, the quality of the remaining bone stock in the trochanteric area and femoral head, the patient’s age and functional demands, the ease of removing broken implants and the surgeon’s criteria. While we agree with this strategy for revision surgery, we consider that (1) although many ingenious methods of implant removal have been reported [8, 18, 28], partial removal of the IM nail is sufficient for patients whose fractures have healed unless the patient strongly refuses this method; (2) in osteosynthesis revision, which kind of internal fixation is performed should be determined on the basis of the orthopaedic surgeon’s familiarity with the procedure of specific fixation (Fig. 4). The priority of IM nails is reduced in osteosynthesis revision due to an extensive incision made for the removal of broken nails and bone allograft at the non-union site, additional cerclage wiring or plate implantation performed to assist reduction, and the disability of IM nails in stabilizing the allograft bone. Furthermore, no significant differences were found in the re-failure rates between the types of IM nails and EM devices (Table 5). In the 3 cases that underwent osteosynthesis revision in our study, 2 were revised with plates, and one was revised with nails, and all of them healed.

**Fig. 5:**
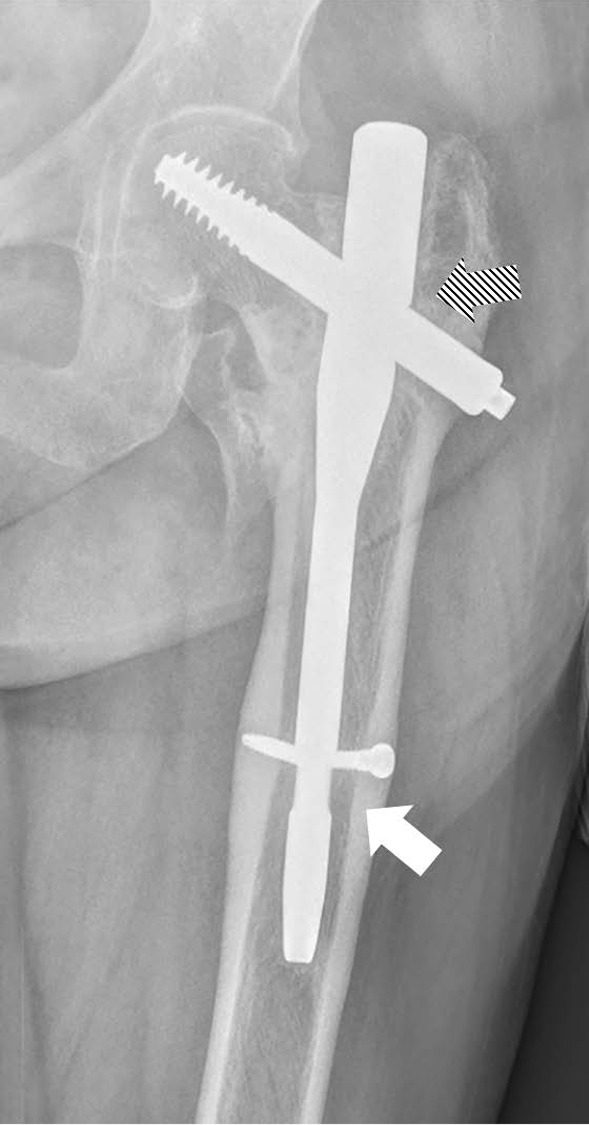
A case of self-dynamism: breakage of the distal screw (white arrow) followed by a fracture of the nail (shadow arrow)

Due to the difficulty of managing a broken nail in revision surgery, it is favourable for orthopaedic surgeons to recognize patients prone to this complication and avoid nail breakage. Self-dynamisation (Fig. 5) occurs after the breakage of distal screws, and its role in the process of nail breakage is still controversial [7, 14]. We consider it a manifestation of poor reduction and difficulty in bone healing. However, the effect of compression caused by self-dynamisation is often extremely limited [5, 12], and fibrous soft tissue can grow into the fracture gap before it occurs. Thus, it is useless in promoting fracture healing and avoiding nail breakage [5, 14]. In fact, self-dynamisation was observed in 17.1% (12/58) of the included cases, we hence consider it a warning sign of nail breakage and an indication for osteosynthesis revision. We also agree with some authors’ recommendation that revision osteosynthesis should be considered at 6 months for patients with persistent pain, suboptimal reduction without consolidation signs [7].

This is a retrospective study and has inherent limitations. Although 70 cases were included in the cohort, the total study period was more than 25 years. Furthermore, comparative analysis could not be performed due to the absence of a control group. Thus, all the conclusions should be interpreted with caution.

In conclusion, intramedullary nail breakage is a rare complication that mostly occurs in unstable pertrochanteric fractures with suboptimal reduction, the unsatisfactory placement of IM nails and delayed union/non-union. It has a relatively late average occurrence time, but lacks of time regularity. Revision surgery needs to be individualized to manage broken nails. Osteosynthesis revision can be conduct by a new IM nail or EM device but considerable secondary failure rate (15.2%) is noteworthy. Self-dynamisation may be a warning sign of nail breakage and an indication for revision osteosynthesis as well.

## Data Availability

The datasets used and/or analysed during the current study are available from the corresponding author on reasonable request.

## Abbreviations

IM: Intramedullary
EM: Extramedullary
AO/OTA: Arbeitsgemeinschaft für Osteosynthesefragen/Orthopaedic Trauma Association
LCP: Locking compression plate
PFN: Proximal femoral nail
PFNA: Proximal femoral nail antirotation
PFNA2: Proximal femoral nail antirotation for Asia
PFP: Proximal femur locking plate
THA: Total hip arthroplasty
TFN: Trochanteric fixation nail
TFNA: Trochanteric fixation nail-advanced
